# Comparison of Amplicon-Based Next-Generation Sequencing Testing and Immunohistochemical Staining in Detecting *Anaplastic Lymphoma Kinase* Fusion Genes in Non-Small-Cell Lung Cancer: A Large Single-Centre Cohort Study

**DOI:** 10.3390/cancers18132125

**Published:** 2026-06-30

**Authors:** Yuichiro Suzukawa, Yuto Tagawa, Seigo Katakura, Shuhei Teranishi, Tetsuro Kondo, Haruhiro Saito, Shuji Murakami

**Affiliations:** Department of Thoracic Oncology, Kanagawa Cancer Center, 2-3-2 Nakao, Asahi-ku, Yokohama 241-8515, Kanagawa, Japan; yr.suzukawa.med@gmail.com (Y.S.);

**Keywords:** *anaplastic lymphoma kinase*, immunohistochemistry, next-generation sequencing, non-small-cell lung cancer, precision medicine

## Abstract

**Background/Objectives:** *Anaplastic lymphoma kinase* (*ALK*) fusion is a driver gene translocation detected in 3–5% of patients with non-small-cell lung cancer (NSCLC). Next-generation sequencing (NGS)-based tests are the standard of care for detecting actionable gene alterations; however, false negatives remain a concern. Immunohistochemical staining is another reliable, rapid, and low-cost method for detecting *ALK* fusions. Previous studies have reported high concordance with NGS, although further studies are needed to draw definitive conclusions. **Methods:** A retrospective analysis was conducted on consecutive patients with NSCLC who were tested using the Oncomine Dx Target Test (ODxTT), an amplicon-based DNA and RNA NGS test for NSCLC, and ALK-immunohistochemistry (IHC) at our institution between 8 August 2019 and 11 April 2025. **Results:** Of 919 eligible patients included in this study, *ALK* fusion was detected in 30 (3.26%) patients, whereas ALK-IHC was positive in 35 (3.80%) patients. The concordance and κ coefficient of the two tests were 99.4% and 0.920, respectively. The sensitivity, specificity, positive predictive value, and negative predictive value of ALK-IHC for ODxTT were 100%, 99.4%, 85.7%, and 100%, respectively. Five discordant patients were NGS negative and IHC positive. Among the five discordant cases, one had a false-negative NGS result, whereas the remaining four had false-positive ALK-IHC results, including three patients with neuroendocrine carcinomas. **Conclusions:** ALK-IHC shows diagnostic accuracy comparable to ODxTT, although prudent interpretation is needed for patients without adenocarcinoma. Our findings suggest the complementary role of ALK-IHC alongside NGS-based testing, particularly in patients with a high pre-test probability of harbouring *ALK* fusions.

## Simple Summary

Comprehensive next-generation sequencing (NGS) has become the standard approach for the identification of actionable driver alterations in non-small-cell lung cancer (NSCLC). However, amplicon-based RNA sequencing may fail to detect *ALK* fusion genes in a small subset of cases because of technical limitations. In this study, we retrospectively compared ALK immunohistochemistry (ALK-IHC) with amplicon-based NGS in 919 patients with NSCLC and confirmed the high concordance between the two methods. Importantly, one clinically significant *ALK* fusion was missed by amplicon-based NGS but was detected by ALK-IHC and confirmed by hybrid-capture sequencing. Our findings also confirmed previous observations that pulmonary neuroendocrine carcinoma may exhibit false-positive ALK-IHC staining in the absence of *ALK* rearrangements, highlighting an important diagnostic pitfall. These results support the selective use of ALK-IHC as a complementary test in patients with a high pre-test probability of *ALK* rearrangement or its use after multiplex molecular testing failure.

## 1. Introduction

Since the discovery of the *EML4–ALK* fusion gene in NSCLC in 2007, *ALK* rearrangements have been established as oncogenic driver alterations and are detected in approximately 3–5% patients with non-small-cell lung cancer (NSCLC) [1,2]. *ALK*-rearranged NSCLC is associated with distinct clinicopathological characteristics: patients are typically younger and have little or no smoking history, and most tumours are classified as adenocarcinoma [3]. *ALK* rearrangements are rare in squamous cell carcinoma and other non-adenocarcinoma histologies [4,5]. The introduction of tyrosine kinase inhibitors (TKIs) for the treatment of advanced NSCLC harbouring *ALK* rearrangements has dramatically improved patient prognosis [6,7]. In addition, their application has expanded to include locally advanced disease [8,9]. In contrast, immune checkpoint inhibitors, another major therapeutic modality in modern oncology, are largely ineffective in *ALK*-fusion-positive NSCLC [10,11]. Therefore, the identification of all patients harbouring this translocation is critically important.

Traditionally, *ALK* fusion was detected using fluorescence in situ hybridisation. More recently, the D5F3 rabbit monoclonal antibody, a highly sensitive and specific immunohistochemical clone, has become an established diagnostic tool [12,13,14]. However, with the growing number of genes to be screened, next-generation sequencing (NGS)-based multigene panel testing has emerged as the standard frontline testing approach because of its comprehensive coverage and efficiency compared with sequential testing [15,16]. Nevertheless, previous reports suggest that the success rate of NGS assays is approximately 80–90% [17,18]. Amplicon-based NGS assays require a relatively large amount of specimen and may fail to detect fusions with unknown partners [19], leading to false-negative results. Therefore, ALK-immunohistochemistry (IHC) may remain a valuable diagnostic modality, offering advantages in terms of speed, sensitivity, minimal specimen requirements, and cost. However, an important pitfall is that ALK protein expression has occasionally been reported in pulmonary neuroendocrine carcinomas in the absence of underlying *ALK* rearrangements, highlighting the need for the cautious interpretation of ALK-IHC results in these tumours [20].

Previous studies have reported sensitivities and specificities of ALK-IHC, based on various NGS tests, of 75.0–92.8% and 98.7–99.9%, respectively [21,22,23]. Zhao et al. [21] compared hybrid-capture DNA NGS and RNA NGS with ALK-IHC and identified 62 discordant cases. In that study, 13 patients received ALK-TKIs, with response rates of 85.7% (6/7) and 66.7% (4/6) in NGS-positive/IHC-negative and NGS-negative/IHC-positive groups, respectively. Wakuda et al. [22] also reported a favourable response to alectinib in a patient with NGS-negative/IHC-positive results. Despite these findings, large cohort studies comparing amplicon-based NGS, which remains indispensable due to its comprehensive coverage and rapid turnaround, and ALK-IHC are limited. Therefore, we aimed to evaluate the diagnostic performance and concordance of ALK-IHC compared with amplicon-based NGS for detecting *ALK* fusion in patients with NSCLC.

## 2. Materials and Methods

### 2.1. Participants

A retrospective analysis was conducted on consecutive patients with pathologically diagnosed NSCLC who were tested using the Oncomine Dx Target Test (ODxTT) (Thermo Fisher Scientific, Waltham, MA, USA) and ALK-IHC at our institute between 8 August 2019 and 11 April 2025. ODxTT is an amplicon-based DNA and RNA NGS and a standard multiplex genetic test for NSCLC covered by Japanese universal health insurance.

### 2.2. NGS

The specimens were sent to SRL Inc. (Tokyo, Japan) for ODxTT testing. Experienced pathologists carefully selected the specimens in accordance with the ODxTT user guide, the Guidelines for the Handling of Pathological Tissue Specimens for Genomic Medicine by Japanese Society of Pathology [24], and specimen requirements defined by SRL, Inc. As stated in the ODxTT user guide, the tumour content must be >30%, and when it is 10–30%, microdissection is necessary. In such cases, the tissue area was marked on formalin-fixed paraffin-embedded (FFPE) slides at our institute, and SRL, Inc. performed microdissection. For specimens <2 mm^2^, multiple FFPE blocks were combined to form a specimen area of ≥2 mm^2^ before submission or multiple slides were prepared from a single FFPE block to achieve a specimen area of ≥2 mm^2^; up to 15 FFPE slides were submitted. An RNA concentration of ≥1.43 ng/µL was required for analysis. According to the ODxTT user documentation, samples may be reported as invalid or no-call when insufficient material is available or when quality-control criteria for nucleic-acid integrity, library preparation, sequencing performance, or downstream analysis are not met. Herein, cases reported as RNA analysis failure via ODxTT were classified as test failures and were excluded from sensitivity and specificity calculations.

### 2.3. Immunohistochemical Staining

The IHC reagent used was a Ventana OpticView ALK (D5F3) rabbit monoclonal antibody (Ventana Medical Systems, Tucson, AZ, USA). A Ventana OpticView DAB universal kit (Ventana Medical Systems) was used for visualisation. Staining was performed at our institute using a Ventana BenchMark Ultra automated immunostainer (Ventana Medical Systems) following the manufacturer’s protocol. Some of the lung resection specimens were stained with the NCL-L-ALK (5A4) mouse monoclonal antibody (Leica Biosystems, Newcastle upon Tyne, UK) followed by a visualisation process performed using the Leica Novolink Polymer Detection System (Leica Biosystems). These specimens were processed using a histostainer (Nichirei Bioscience Inc., Tokyo, Japan), following the manufacturer’s protocol.

### 2.4. Statistical Analysis

Statistical analyses were performed using Python version 3.12.12. Python libraries, including Statsmodels [25] and scikit-learn [26], were used. Agreement between IHC and ODxTT results was assessed using concordance and Cohen’s κ coefficient, with 95% confidence intervals (CIs) estimated via a nonparametric bootstrap method with 50,000 resamples. The sensitivity, specificity, positive predictive value (PPV), and negative predictive value (NPV) were calculated using ODxTT as the reference standard, given its widespread use in Japanese clinical practice. The 95% CIs were calculated using the Clopper–Pearson method.

### 2.5. Ethics

The requirement for written informed consent was waived because of the retrospective nature of the study. An opt-out approach was used, and information concerning the study and the secondary use of clinical data was publicly communicated on our hospital website (https://kcch.kanagawa-pho.jp/data/media/kanagawa-hospital/page/pdf/cr-kokyukinaika/op2025eki24a.pdf, accessed on 28 June 2026). This study was approved by the Institutional Review Board, Kanagawa Cancer Center (approval number: 2025 eki-24) and was conducted in accordance with the guidelines of the Declaration of Helsinki.

### 2.6. Declaration of Generative AI and AI-Assisted Technologies in the Manuscript Preparation Process

During the preparation of this work, the authors used ChatGPT ver. 5.2 (OpenAI, San Francisco, CA, USA) for the literature review, English-language draft preparation, and Python code development. The authors also used OpenEvidence (https://www.openevidence.com/; OpenEvidence Inc., Cambridge, MA, USA) for the literature review. After using this tool, the authors reviewed and edited the content as needed and take full responsibility for the content of the published article.

## 3. Results

### 3.1. Patient Characteristics

**Figure 1:**
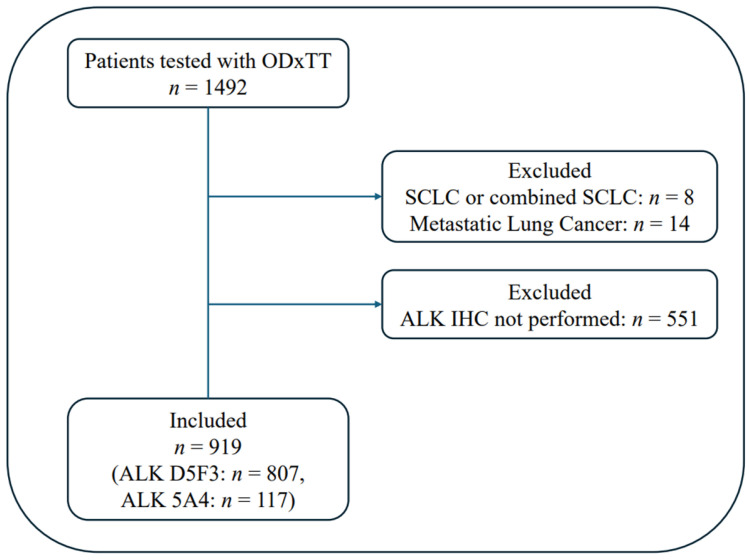
CONSORT flow diagram of the study.

During the study period, 1492 patients were tested using ODxTT. Eight patients were diagnosed with small cell lung cancer and 14 with pulmonary metastases from other primary tumours after a thorough pathological review; these patients were therefore excluded from the analysis. Among the 1470 included patients, 919 underwent ALK-IHC testing. A total of 807 patients (87.8%) were tested using ALK (D5F3), with 117 (12.7%) using ALK (5A4). Five patients were tested with both D5F3 and 5A4 antibodies, and all were positive based on both assays. A CONSORT flow diagram is shown in Figure 1. Most patients in the present cohort were selected from two clinical pathways. First, since May 2021, routine testing with both ODxTT and ALK-IHC (D5F3) has been performed for patients with stage IV NSCLC managed primarily by the Department of Thoracic Oncology. Second, ALK-IHC (5A4) screening was performed for a subset of surgically resected cases as part of a separate research study. Together, these groups accounted for most patients included in the present analysis (Tables S3 and S4).

Patient characteristics are summarised in Table 1. The median age was 72 (range, 29–93) years, and 298 patients (32.4%) were female. A total of 210 patients (22.9%) were never smokers, and 569 (61.9%) had adenocarcinoma. Although race was not recorded, the cohort was presumed to be predominantly of Japanese heritage based on the sociodemographic characteristics of patients referred to our institution.

Specimen types are listed in Table 2. Biopsy accounted for most sampling methods (80.1%), with transbronchial lung biopsy (TBLB) being the most commonly used technique (51.0%).

### 3.2. Concordance Between ODxTT and ALK-IHC

The ODxTT success rates for DNA, RNA, and both components were 98.0%, 98.3%, and 96.5%, respectively. All 15 cases of ODxTT RNA NGS failure tested negative via ALK-IHC and were excluded from the diagnostic performance analysis. *ALK* fusion was detected using ODxTT in 30 patients (3.26%), whereas ALK-IHC was positive in 35 patients (3.80%). Concordance and the κ coefficient of the two tests were 99.4% (95% CI: 98.7–99.8%) and 0.920 (95% CI: 0.838–0.982), respectively. The sensitivity, specificity, PPV, and NPV of ALK-IHC referred to ODxTT were 100% (95% CI: 88.4–100%), 99.4% (95% CI: 98.6–99.8%), 85.7% (95% CI: 69.7–95.1%), and 100% (95% CI: 99.5–100%), respectively (Table 3). A subgroup analysis of 569 patients with adenocarcinoma yielded higher concordance and ALK-IHC accuracy: concordance/κ coefficient = 99.6% (95% CI: 98.7–99.9%)/0.964 (95% CI: 0.901–1.000); and sensitivity/specificity/PPV/NPV = 100% (95% CI: 87.6–100%)/99.6% (95% CI: 98.6–99.9%)/93.3% (95% CI: 77.9–99.1%)/100% (95% CI: 99.3–100%) (Table S1).

### 3.3. Discordant Patients

**Figure 2:**
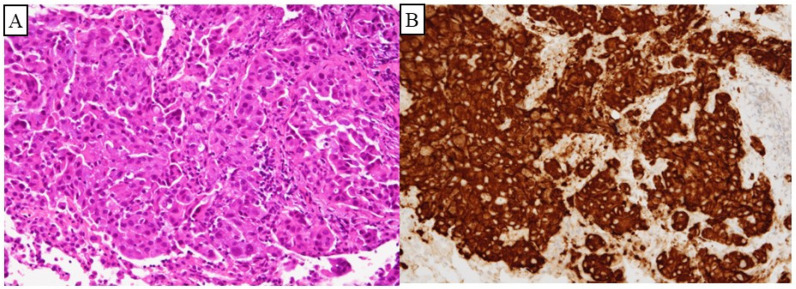
HE and ALK-IHC image of discordant Patient 1. (**A**) Adenocarcinoma HE staining, ×200; (**B**) adenocarcinoma ALK (D5F3) IHC positive, ×200; HE, haematoxylin and eosin; ALK-IHC; anaplastic lymphoma kinase-immunohistochemistry.

All five discordant patients were NGS-negative/IHC-positive (Table 4). Patient 1 was a young, never-smoking patient with adenocarcinoma who showed a marked response to first-line lorlatinib and remained on treatment 19 months later. Pathological images are shown in Figure 2, demonstrating the diffuse strong cytoplasmic staining typically observed in *ALK*-rearranged tumours. TrueSight Oncology 500 (TSO500; Illumina, Inc., San Diego, CA, USA), a hybrid capture-based DNA and RNA NGS assay, detected *EML4-ALK* (E13; A20), confirming a false-negative result via ODxTT. The same specimen was used for both NGS tests, and the inaccurate ODxTT result was attributed to RNA degradation and limitations of the amplicon-based method.

Patient 2 was a young, never-smoking patient with adenocarcinoma. ODxTT detected a *BRAF* V600E mutation but no *ALK* fusion. First-line treatment with dabrafenib and trametinib was initiated, resulting in a favourable response that lasted 4 months. An experienced pathologist (blinded to clinical information) reviewed the ALK-IHC slide and, based on several objective histopathological findings, interpreted it as exhibiting non-specific ALK staining. First, staining was observed on the ALK-IHC negative control slide. Second, ALK immunoreactivity was detected in non-neoplastic elements, including inflammatory cells and normal bronchial epithelial cells. Third, tumour cells showed a heterogeneous staining pattern rather than the diffuse strong cytoplasmic staining typically observed in *ALK*-rearranged tumours.

Patients 3–5 had neuroendocrine carcinomas (NECs). The underlying mechanism remains unclear but may reflect the aberrant expression of wild-type *ALK* rather than a true *ALK* rearrangement. Immunohistochemical findings supporting the neuroendocrine phenotype are shown in Table S2. Of note, patient 3 was initially diagnosed with poorly differentiated NSCLC, not otherwise specified, and treatment with alectinib was initiated; however, the patient died within 2 weeks due to disease progression. A subsequent detailed pathological evaluation confirmed a diagnosis of NEC.

## 4. Discussion

The success rate of ODxTT in the present study was higher than previously reported. After initially experiencing low success rates at our institution, we implemented stricter specimen quality control, which led to substantial improvement. In 2019, the success rate for DNA and RNA analyses using ODxTT was as low as 75.6%. Following these changes, the success rate increased to 96.0% during 2020–2025. Although ODxTT is a reliable assay when high-quality specimens are available, a proportion of test failures persists and may affect clinical decision-making.

In routine clinical practice in Japan, repeat submission for NGS testing after an ODxTT failure is uncommon. First, repeat NGS testing is not reimbursed under the national health insurance system. Second, the resubmission of the same specimen is unlikely to be informative because the underlying causes of failure, such as insufficient nucleic acid quality or quantity, often remain unchanged. Re-biopsy is another potential option but may not be justified because of its invasiveness and the absence of any guarantee that the newly obtained specimen will successfully undergo NGS analysis. In selected patients with a particularly high pre-test probability of harbouring actionable driver alterations such as never-smokers with adenocarcinoma, alternative low-input single-gene assays, including *EGFR* PCR-based mutation testing or ALK-IHC, may be considered. Re-biopsy may also be pursued on a case-by-case basis after the careful consideration of the patient’s clinical condition, procedural risks, and anticipated impact on treatment selection.

In this cohort, RNA analysis failed in 15 patients (1.6%). Detailed laboratory-level causes of failure were not available because testing was performed at an external reference laboratory. According to the ODxTT user documentation, invalid or no-call results may occur because of insufficient sample material or failure to satisfy assay quality-control requirements. All 15 cases tested negative via ALK-IHC, and none were subsequently diagnosed as *ALK*-rearranged NSCLC during follow-up.

Previous studies comparing NGS-based testing with ALK-IHC have reported high concordance, with sensitivities and specificities of 75.0–92.8% and 98.7–99.9%, respectively [21,22,23]. In our study, the sensitivity and specificity of ALK-IHC were 100% (95% CI, 88.4–100%) and 99.4% (95% CI, 98.6–99.8%), respectively, further supporting its reliability.

A key strength of this study is the comparison of ALK-IHC with amplicon-based NGS testing. This differs from the study by Zhao et al. [21], which evaluated hybrid capture-based DNA and RNA NGS, methods that are less commonly used as first-line tests due to higher costs and a longer turnaround time. In addition, our study incorporated clinical outcome data, which was not included in the study by Ilié et al. [23]. Furthermore, our cohort size exceeded that reported by Wakuda et al. [22].

The relatively low PPV of 85.7% (95% CI, 69.7–95.1%) was largely attributable to the inclusion of NECs, which comprised 2.1% (19/919) of the cohort. Aberrant ALK overexpression has been reported in pulmonary NECs and is regarded as a potential pitfall when implementing ALK-IHC in the molecular diagnosis of lung cancers [20]. Approximately 90% of ALK-IHC-positive pulmonary NECs have been reported to lack underlying *ALK* rearrangements, suggesting that most positive results represent false-positive staining rather than true *ALK* fusions [27]. Therefore, in the three discordant NEC cases included in the present cohort, it is reasonable to regard these tumours as being *ALK* fusion-negative considering the negative NGS findings. At our institution, ALK-IHC was routinely performed even when neuroendocrine tumour histology was suspected based on haematoxylin and eosin staining, aiming to reduce the turnaround time via the simultaneous staining with neuroendocrine markers such as synaptophysin and chromogranin. However, distinguishing NEC from poorly differentiated NSCLC can be challenging, particularly in small biopsy specimens. Patient 3 illustrates the need for caution when interpreting ALK-IHC in poorly differentiated carcinomas. We performed subgroup analyses in cases of adenocarcinoma because *ALK* rearrangements occur predominantly in adenocarcinoma and represent the most clinically relevant population for ALK testing, resulting in the improved PPV of 93.3% (95% CI, 77.9–99.1%), with only two discordant cases.

Notably, ALK-IHC identified one false-negative ODxTT result (Patient 1). False-negative results in amplicon-based RNA NGS can arise from two main mechanisms. First, this method is designed to detect known fusion partners and breakpoints and may miss fusions involving unknown partners or atypical breakpoints; although a 3′–5′ imbalance score may suggest such fusions, its role is supplementary [19]. Second, RNA quality is critical, and degradation may occur during pre-analytical processing. DV200 is commonly used to assess the RNA quality, with TSO500 requiring a minimum DV200 of >20%. ODxTT requires a minimum RNA concentration of 1.43 ng/µL but does not specify a DV200 threshold. However, a DV200 < 30% is associated with significantly lower success rates and total RNA reads [28] and may serve as a practical reference. Importantly, DV200 reflects overall RNA integrity and does not provide information on specific transcripts. In Patient 1, despite a common fusion variant, an RNA concentration of 6.3 ng/µL, and a DV200 of 39.95%, ODxTT failed to detect the fusion. The DNA NGS component also reported insufficient material for nine genes. Therefore, undetected RNA degradation was considered the most likely cause of the false-negative result.

**Figure 3:**
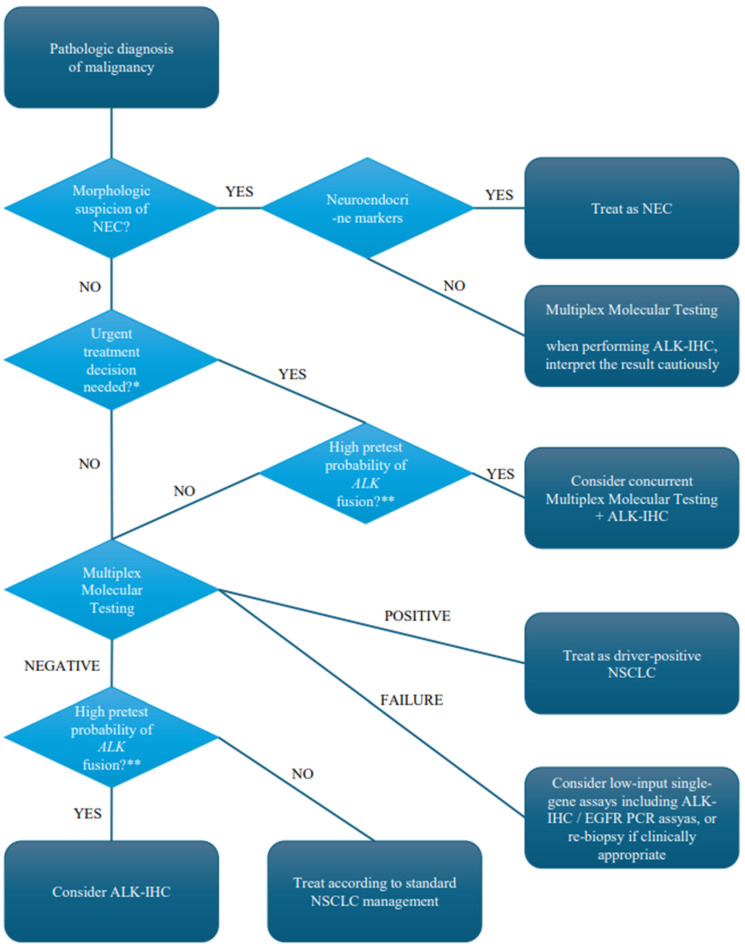
Proposed clinical algorithm for integrating ALK-IHC and multiplex molecular testing in NSCLC. This algorithm presents the diagnostic strategy proposed on the basis of the present findings and should not be interpreted as a clinical practice guideline. * poor PS, high tumour burden, oncologic emergency, etc. ** adenocarcinoma, never/light smoker, young age, or NSCLC-NOS. ALK-IHC, anaplastic lymphoma kinase-immunohistochemistry; NSCLC, non-small-cell lung cancer; NEC, neuroendocrine carcinoma.

This study highlights the technical limitations of amplicon-based RNA NGS and a potential complementary role of IHC testing. Comprehensive genomic profiling using hybrid capture-based assays may identify such cases at a later stage; however, patients with *ALK* rearrangement positive NSCLC derive greater benefit from first-line ALK-TKI therapy [29,30]. Considering the relatively high prevalence of *ALK* fusions among never-smokers with adenocarcinoma (9.8% (18/184) in this cohort) and although multiplex molecular testing remains the primary strategy, ALK-IHC may be used selectively as a complementary test in cases of high pre-test probability or multiplex molecular test failures. Our proposed testing strategy is presented in Figure 3.

This study has some limitations. First, ODxTT was used as the reference standard. Patient 1 was subsequently confirmed as harbouring an *EML4-ALK* fusion via hybrid-capture NGS, demonstrating that ODxTT itself is not a perfect surrogate for the biological *ALK* status. Therefore, the reported sensitivity and specificity values should be interpreted as in agreement with ODxTT rather than as the absolute diagnostic performance.

Second, not all discordant cases underwent orthogonal confirmation testing. Consequently, the definitive determination of the true molecular status was not possible in all cases. Although Patient 2 was considered to most likely to represent non-specific ALK staining, we cannot entirely exclude the rare coexistence of a *BRAF* mutation and an *ALK* fusion. Similarly, although Patients 3–5 were interpreted as exhibiting aberrant ALK expression in neuroendocrine carcinoma, the presence of true *ALK* rearrangements cannot be completely ruled out.

Third, as this was a retrospective, single-centre study, the potential for unmeasured selection bias cannot be excluded. However, following the implementation of routine ALK-IHC testing in the Department of Thoracic Oncology in May 2021, 85.1% of the study cohort comprised consecutive patients evaluated under a standardised testing strategy wherein both ODxTT and ALK-IHC (D5F3) were routinely requested for patients with stage IV NSCLC. In addition, 12.7% of the cohort comprised surgically resected cases that underwent ALK-IHC (5A4) screening according to a predefined institutional research protocol rather than according to physician discretion. Therefore, the present study is unlikely to have been substantially affected by physician-driven test selection bias, although the generalisability of our findings should be interpreted with caution.

Fourth, not all patients were tested using the U.S. Food and Drug Administration–approved ALK (D5F3) companion diagnostic assay. Although one study reported that the ALK (5A4) clone has comparable accuracy to D5F3 for detecting *ALK* fusions [31], another found that 5A4 exhibited a higher false-negative rate [32]. Notably, in our cohort, the concordance between ODxTT and ALK (5A4) was 100% (95% CI: 96.7–100%), including eight concordant-positive and 101 concordant-negative cases, whereas the concordance between ODxTT and ALK (D5F3) was 99.4% (95% CI: 98.6–99.4%) (detailed analyses are presented in Tables S3 and S4). When diagnostic accuracy was evaluated separately using ODxTT as the reference standard, ALK-IHC performed using the D5F3 antibody appeared to show slightly lower diagnostic performance than that performed using the 5A4 antibody. However, this apparent difference should be interpreted with caution because the two antibodies were used in different clinical settings. The 5A4 antibody was used exclusively in surgical specimens; the D5F3 antibody was used predominantly in small biopsy specimens and in cases where neuroendocrine carcinoma could not be confidently excluded based on morphology. Furthermore, the relatively small number of cases in the 5A4 cohort limits a meaningful comparison between the two antibodies. Taken together, these findings suggest that the apparent differences are more likely to reflect differences in specimen characteristics and case selection than intrinsic differences between antibody clones.

## 5. Conclusions

ALK-IHC demonstrated high diagnostic accuracy compared with ODxTT, although careful interpretation is required in patients without adenocarcinoma. Importantly, ALK-IHC identified one positive case that was not detected using ODxTT. Therefore, our findings suggest the complementary role of ALK-IHC alongside NGS-based testing, particularly in patients with a high pre-test probability of harbouring *ALK* fusions.

## Figures and Tables

**Table 1:** Patient demographics (*n* = 919).

Characteristics	*n* = 919
Age, year	
Median (range)	72 (29–93)
Sex	
Female	298 (32.4%)
Male	621 (67.6%)
Smoking status	
Never	210 (22.9%)
Former	493 (53.6%)
Current	214 (23.3%)
Missing	2 (0.2%)
Histology	
Ad	569 (61.9%)
NOS	69 (7.5%)
Sq	236 (25.7%)
NET/NEC	19 (2.1%)
Others	26 (2.8%)

Ad, adenocarcinoma; NOS, NSCLC not otherwise specified; Sq, squamous cell carcinoma; NET, neuroendocrine tumour; NEC, neuroendocrine carcinoma; ‘others’ contain mucinous adenocarcinoma, adeno-squamous carcinoma, and pleomorphic carcinoma.

**Table 2:** Specimen types (*n* = 919).

Characteristics	*n* = 919
Biopsy	736 (80.1%)
TBLB	469 (51.0%)
EBB	31 (3.4%)
EBUS-TBNA	169 (18.4%)
CTGB	43 (4.7%)
PFCB	3 (0.3%)
Others	21 (2.3%)
Surgery	183 (19.9%)

TBLB, transbronchial lung biopsy; EBB, endobronchial biopsy; EBUS-TBNA, endobronchial ultrasound-guided transbronchial needle aspiration; CTGB, computed-tomography-guided biopsy; PFCB, pleural fluid cell block; ‘others’ contain biopsies of lymph node, pancreas, bone, liver, and kidney.

**Table 3:** Concordance of ODxTT and ALK-IHC: all patients (*n* = 919).

		ODxTT		
		Positive	Negative	Failure
ALK-IHC	positive	30	5	0
	negative	0	869	15

Concordance 99.4% (95% CI: 98.7–99.8%); κ coefficient 0.920 (95% CI: 0.838–0.982); sensitivity 100% (95% CI: 88.4–100%); specificity 99.4% (95% CI: 98.6–99.8%); PPV 85.7% (95% CI: 69.7–95.1%); NPV 100% (95% CI: 99.5–100%). ALK-IHC, anaplastic lymphoma kinase-immunohistochemistry; CI, confidence interval; PPV, positive predictive value; NPV, negative predictive value.

**Table 4:** Clinicopathological characteristics of patients with discordant ALK-IHC and ODxTT results.

Patient	Age	Sex	Smoking Status	SI	ALK-IHC	ODxTT Result	Histology	Cause of Discordance	ALK-TKI	Response	TTF
1	46	M	Never	0	Positive	None detected	Ad	ODxTT false-negative	Lorlatinib	PR	19 months+
2	54	M	Never	0	Positive	*BRAF* V600E	Ad	ALK-IHC non-specific staining	None	-	-
3	70	M	Current	1000	Positive	None detected	NEC	False-positive ALK-IHC in NEC	Alectinib	PD	2 weeks
4	53	M	Current	900	Positive	None detected	LCNEC	False-positive ALK-IHC in NEC	None	-	-
5	73	M	Current	580	Positive	None detected	NEC	False-positive ALK-IHC in NEC	None	-	-

SI, smoking index; ODxTT, Oncomine Dx Target Test; TTF, time to treatment failure; M, male; Ad, adenocarcinoma; NEC, neuroendocrine carcinoma; LCNEC, large-cell neuroendocrine carcinoma; PR, partial response; PD, progressive disease. - indicates not applicable because no ALK-TKI was administered. None detected indicates that no actionable genomic alterations were detected by ODxTT.

## Data Availability

Our data are unsuitable to post because they include confidential information such as patient data and other ongoing studies. However, we will provide curated anonymised data upon reasonable requests.

## Supplementary Materials

The following supporting information can be downloaded at: https://www.mdpi.com/article/10.3390/cancers18132125/s1, Table S1: Concordance of ODxTT and ALK-IHC in patients with adenocarcinoma (*n* = 569); Table S2: Immunohistochemical profiles for the diagnosis of neuroendocrine carcinoma.; Table S3: Concordance of ODxTT and ALK-IHC (D5F3) (*n* = 807); Table S4: Concordance of ODxTT and ALK-IHC (5A4) (*n* = 117).

## Abbreviations

The following abbreviations are used in this manuscript:

*ALK*	*Anaplastic lymphoma kinase*
FFPE	Formalin-fixed paraffin-embedded
IHC	Immunohistochemistry
NGS	Next-generation sequencing
NPV	Negative predictive value
NSCLC	Non-small-cell lung cancer
ODxTT	Oncomine dx Target Test
PPV	Positive predictive value
TBLB	Transbronchial lung biopsy
TKIs	Tyrosine kinase inhibitors
