# Supplementary material for: Comparison of Amplicon-Based Next-Generation Sequencing Testing and Immunohistochemical Staining in Detecting Anaplastic Lymphoma Kinase Fusion Genes in Non-Small-Cell Lung Cancer: A Large Single-Centre Cohort Study
Source: Cancers (Basel). 2026 Jun 30;18(13):2125. doi: 10.3390/cancers18132125 (PMC13359545; doi:10.3390/cancers18132125)
Supplement: Supplementary file 1 [file cancers-18-02125-s001.zip › cancers-4386082-supplementary.pdf]

Table S1. Concordance of ODxTT and ALK-IHC: adenocarcinoma patients (n = 569)

		ODxTT		
		Positive	Negative	Failure
ALK-IHC	positive	28	2	0
	negative	0	532	7

Concordance 99.6% (95% CI: 98.7–99.9%); κ coefficient 0.963 (95% CI: 0.901–1.00); sensitivity 100% (95% CI: 87.6–100%); specificity 99.6% (95% CI: 98.6–99.9%); PPV 93.3% (95% CI: 77.9–99.1%); NPV 100% (95% CI: 99.3–100%)
ALK-IHC, anaplastic lymphoma kinase-immunohistochemistry; CI, confidence interval; PPV, positive predictive value; NPV, negative predictive value

Table S2. Immunohistochemical profiles for the diagnosis of neuroendocrine carcinoma

**Patient no.**	**Histology**	**CK AE1/AE3**	**CD56 NCAM**	**Synapto-physin**	**Chromo-granin A**	**RB1**	**Ki-67 index (%)**
**3**	NSCLC-NOS →NEC	+	-	+/-	-	-	≥90%
**4**	LCNEC	+	+	+	+	-	80–90%
**5**	LCNEC	+	+	+	+	+	90%

NSCLC, non-small cell lung cancer; NOS, not otherwise specified; NEC, neuroendocrine carcinoma; LCNEC, large-cell neuroendocrine carcinoma

Table S3. Concordance of ODxTT and ALK-IHC (D5F3) (n = 807)

		ODxTT		
		Positive	Negative	Failure
ALK-IHC	positive	26	5	0
	negative	0	769	7

Concordance 99.4% (95% CI: 98.6–99.4%); κ coefficient 0.909 (95% CI: 0.816–0.980); sensitivity 100% (95% CI: 86.8–100%); specificity 99.4% (95% CI: 98.5–99.8%); PPV 83.9% (95% CI: 66.3–94.5%); NPV 100% (95% CI: 99.5–100%)
ALK-IHC, anaplastic lymphoma kinase-immunohistochemistry; CI, confidence interval; PPV, positive predictive value; NPV, negative predictive value

Table S4. Concordance of ODxTT and ALK-IHC (5A4) (n = 117)

		ODxTT		
		Positive	Negative	Failure
ALK-IHC	positive	8	0	0
	negative	0	101	8

Concordance 100% (95% CI: 96.7–100%); κ coefficient 1.00, indicating perfect agreement*; sensitivity 100% (95% CI: 63.1–100%); specificity 100% (95% CI: 96.4–100%); PPV 100% (95% CI: 63.1–100%); NPV 100% (95% CI: 96.4–100%)

ALK-IHC, anaplastic lymphoma kinase-immunohistochemistry; CI, confidence interval; PPV, positive predictive value; NPV, negative predictive value

*) A 95% CI for κ was not estimated because no discordant cases were observed and bootstrap resampling occasionally produced degenerate 2×2 tables
